# Effects of Side-Chain Engineering with the S Atom in Thieno[3,2-*b*]thiophene-porphyrin to Obtain Small-Molecule Donor Materials for Organic Solar Cells

**DOI:** 10.3390/molecules26206134

**Published:** 2021-10-11

**Authors:** Liuping Xie, Wei Tang, Zhixin Liu, Wencheng Tang, Zihao Yuan, Yinbin Qin, Lei Yan, Xunjin Zhu, Weiguo Zhu, Xingzhu Wang

**Affiliations:** 1 School of Physics and Optoelectronics, College of Chemistry, Xiangtan University, Xiangtan 411105, China; Xieliuping@126.com (L.X.); m15367577269@163.com (W.T.); 18216409205@163.com (Z.L.); t1584635430@icloud.com (W.T.); zh_yaun_1226@163.com (Z.Y.); q782237957@163.com (Y.Q.); zhuwg18@126.com (W.Z.); 2 Department of Chemistry and Institute of Molecular Functional Materials, Hong Kong Baptist University, Waterloo Road, Kowloon Tong, Hong Kong, China; xjzhu@hkbu.edu.hk; 3 Academy for Advanced Interdisciplinary Research, Department of Materials Science & Engineering, Southern University of Science and Technology, Shenzhen 518055, China

**Keywords:** thieno[3,2-*b*]thiophene(TT)-substituted porphyrins, thiolalkyl chain, donor, organic solar cells (OSCs), devices

## Abstract

To explore the effect of the introduction of heteroatoms on the properties of porphyrin materials, a new porphyrin-based derivative small-molecule donor named as PorTT-T was designed and synthesized based on alkyl-thieno[3,2-*b*]thiophene(TT)-substituted porphyrins. The linker bridge and end groups of PorTT-T were the same as those of XLP-II small-molecule donor materials, while the side-chain attached to the core of thieno[3,2-*b*]thiophene(TT)-substituted porphyrin was different. Measurements of intrinsic properties showed that PorTT-T has wide absorption and appropriate energy levels in the UV-visible range. A comparison of the morphologies of the two materials using atomic force microscopy showed that PorTT-T has a better surface morphology with a smaller root-mean-square roughness, and can present closer intermolecular stacking as compared to XLP-II. The device characterization results showed that PorTT-T with the introduced S atom has a higher open circuit voltage of 0.886 eV, a higher short circuit current of 12.03 mAcm^−2^, a fill factor of 0.499, a high photovoltaic conversion efficiency of 5.32%, better external quantum efficiency in the UV-visible range, and higher hole mobility.

## 1. Introduction

The severe impact of energy resource depletion and environmental pollution on human health and society has motivated an ongoing search for clean energy sources to replace those of non-renewable origin [1,2,3]. Organic small-molecule photovoltaic cell materials have attracted intense attention due to their importance as the basis of low-cost devices that can be used for the large-scale generation of renewable energy through photovoltaic solar energy conversion [4,5,6,7,8]. Organic small-molecule materials are easier to modify than polymers due to the lower number of synthesis steps, simple molecular structures, and well-defined molecular weights [9,10,11]. Therefore, organic small-molecule materials are generally preferred with respect to polymer materials as organic semiconductor materials in photovoltaic cells [12,13,14]. There are two classical structures for the molecular design of small molecule donor materials: acceptor-donor-acceptor (A-D-A) [15,16,17,18] and donor-acceptor-donor (D-A-D) [19,20,21]. Among these, the A-D-A type of donor material is more popular due to the availability of a wider variety of structures. For example, different donor units can be used as the core, the acceptor units acting as terminal groups can be varied, and at the same time, some conjugated units can be introduced between the acceptor units as linker bridges to form small molecules with the acceptor--π-donor--π-acceptor (A-π-D-π-A) type of structure. For some molecules with large planar conjugated structure such as porphyrins [22] or phthalocyanines [23,24,25], central metal atoms can be introduced or replaced, and heteroatoms [26] can be introduced into their side-chain positions to effectively regulate the molecular energy levels and properties in order to improve the performance of these materials in organic photovoltaic devices.

**Figure 1:**
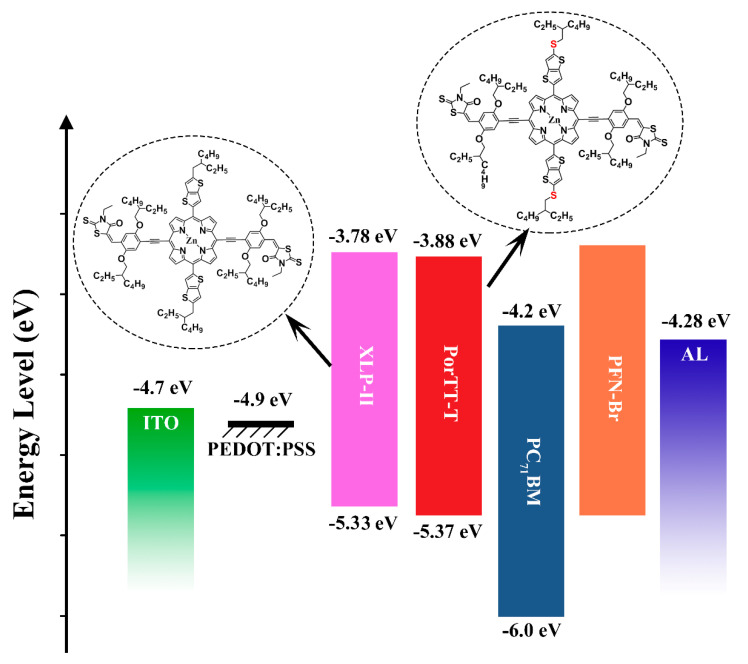
Molecular structure of PorTT-T and XLP-II, and the energy levels of the layers of the device.

In particular, side-chain modification has been widely used as an effective method to regulate the properties of molecular structure in organic photovoltaic materials [27,28,29]. For example, the dissolution and crystallinity of the molecules can be effectively regulated by appropriately lengthening or shortening the length of the alkyl side chains, changing the shape of alkyl chains, and using heteroatom substitution [30,31,32]. In these methods, the effect of heteroatom replacement on the properties of the material is significant. In our previous report [33], the performance of a photovoltaic device based on the XLP-II material (see Figure 1 for the structure) was poor, and atomic force microscopy (AFM) images showed that the performance of the device was mainly due to the unsatisfactory morphology of the films formed by the XLP-II:PC_71_BM blend film. 

To address this problem, in this work we modified the molecular structure of XLP-II and obtained a new type of small-molecule porphyrin named PorTT-T. By introducing a sulfur atom at the point of contact between the porphyrin core and the alkyl side chain, the solubility and performance of the materials are improved. PorTT-T differs from XLP-II only in its central building block, which is composed of branched alkylthio chain-TT-substituted porphyrin and retains the same linker-bridge and same end group in the main structure. The chemical structure of PorTT-T is shown in Figure 1. We adopted a series of different conditions for the processing of the device based on the PorTT-T material and screened different annealing temperatures and solvent post-treatments of the material to optimize device performance. The PCE of the device based on PorTT-T:PC_71_BM was 5.32%, an improvement of approximately 2.2% compared with XLP-II. These results demonstrated that the use of alkylthio chain as the side group instead of the alkyl chain side group can effectively improve the PCE of porphyrin-based derivatives acting as donor materials for small-molecule OPVs.

## 2. Results and Discussions

### 2.1. Optical Absorption Properties of Solutions and Films

**Figure 2:**
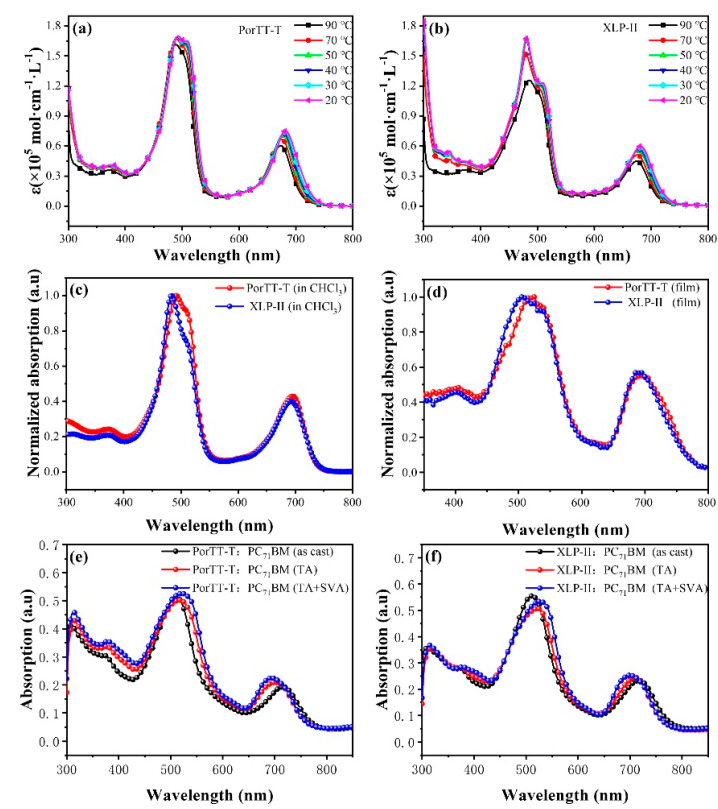
UV-Vis spectra of PorTT-T and XLP-II. (**a**,**b**) Absorption in chlorobenzene (1 × 10^−5^ mol/L) at different temperatures. (**c**) Normalized absorption of PorTT-T and XLP-II in CF and (**d**) absorption of films of PorTT-T and XLP-II. (**e**,**f**) Absorption of films obtained with different post-treatments.

We obtained a series of UV-Visible absorption spectroscopy measurements on PorTT-T and XLP-II. The results are shown in Figure 2. First, we analyzed the properties of the material itself, and examined PorTT-T and XLP-II at a concentration of 1 × 10^−5^ mol/L in chlorobenzene at different temperatures. The spectral absorption diagram of the material for temperatures down from 90 to 20 °C is shown in Figure 2a,b, and the corresponding parameter values are listed in Table 1. We raised the temperature of the solution to 90 °C, and found that the maximum absorption wavelength (λ_max_) was 488 nm, and its corresponding absorption coefficient was 1.61 L/mol cm. When the temperature of the solution was decreased to 70 °C, the maximum absorption spectrum shifted to 489 nm, and the corresponding absorption coefficient increased to 1.67 L/mol cm. In addition, when the solution temperature of XLP-II was raised to 90 °C, the maximum absorption wavelength (λ_max_) was 481 nm, and the corresponding absorption coefficient was 1.61 L/mol cm. When the solution temperature dropped to 20 °C, the maximum absorption spectrum moved to 508 nm, and the corresponding absorption coefficient increased to 1.23 L/mol cm. Thus, it was found that the absorption spectrum redshifts and the absorption coefficient increased with decreasing temperature. The absorption spectrum of XLP-II was more sensitive to the increase in temperature. Figure 2c shows the absorption spectrum changes of the PorTT-T films under three conditions of casting with solvent without any post-treatment (as-cast), thermal annealing only (TA), and both thermal annealing and post-treatment with solvent evaporation annealing (TA + SVA). The maximum absorption wavelengths of the Soret band (400–550 nm) and Q band (600–750 nm) for the PorTT-T films (as-cast) were 509 nm and 717 nm, respectively. After annealing at 100 °C, their maximum absorption wavelengths were 518 nm and 700 nm, respectively. For the film after annealing and solvent post-treatment with dichloromethane, the maximum Soret- and Q-band absorption wavelengths were 522 nm and 694 nm, respectively. The UV-visible spectra clearly showed that the absorption of the post-treated film demonstrated a significant redshift in the Soret-band, while the absorption spectrum of the corresponding Q-band displayed a blueshift. Figure 2c,d, respectively, present the absorption spectra of PorTT-T and XLP-II in solution and the corresponding films, and Table 2 presents the optical data. It could be seen that the PorTT-T spectra were redshifted relative to the XLP-II spectra. For the absorption spectra of the molecules in solution in particular, it was observed that the Soret-band shoulder peak strengthened, showing that the π-π stacking was also strengthened [34] when the S atom was introduced between the molecular core and the alkyl side chain. Due to the introduction of sulfur atoms, the intermolecular forces and the self-assembly ability of the material were effectively improved. An examination of the thin-film absorption spectra showed that the Soret-band of PorTT-T was redshifted by approximately 10 nm compared to XLP-II. The onset of the normalized thin-film optical absorption spectra of PorTT-T was redshifted to 772 nm compared with that of the solution, corresponding to an optical band gap of 1.60 eV. These results show that the substitution of carbon atoms by heteroatoms (as S atom) on the side chain can strongly affect the intermolecular interactions, optical absorption, and optical band gap (E_g_^opt^).

### 2.2. DFT Calculations and Electrochemical Properties

**Figure 3:**
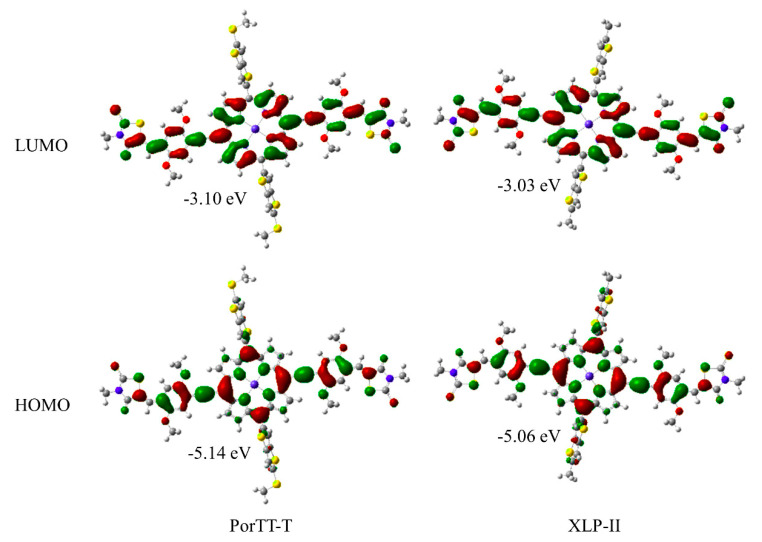
Frontier molecular orbitals of PorTT-T and XLP-II obtained by DFT calculations.

To further understand the three-dimensional geometric structure and molecular orbitals of the S atom pair porphyrin small molecules on the side chain, we performed density functional theory (DFT) calculations for PorTT-T under the B3LYP/6-311G* energy level by the soft of Gaussian-09. Figure 3 and Figure S5 show the front and side views of the frontier molecular orbitals of PorTT-T. According to DFT calculations, the energy of the highest occupied molecular orbital (HOMO) (E_HOMO_^cal^) was −5.14 eV, and the energy of the lowest unoccupied molecular orbital (LUMO) (E_LUMO_^cal^) was −3.10 eV. According to the DFT calculation results, the S atom in the side chain contributed little to the electron density distribution on the LUMO of PorTT-T. However, based on the electron density distribution of the HOMO, the introduction of S atom reduced the electron density on the meso-thieno[3,2-*b*]thiophene(TT)-substituted porphyrins, making the electron density of the HOMO more concentrated on the main chain with porphyrin as the center. At the same time, the S atom led to the dihedral angles of the two planes of thieno[3,2-*b*]thiophene and porphyrin core becoming larger (from 70° to 72°) (Figure S5). The DFT calculation results were similar to the HOMO and LUMO results obtained from CV experimental data, and the trend was consistent.

**Figure 4:**
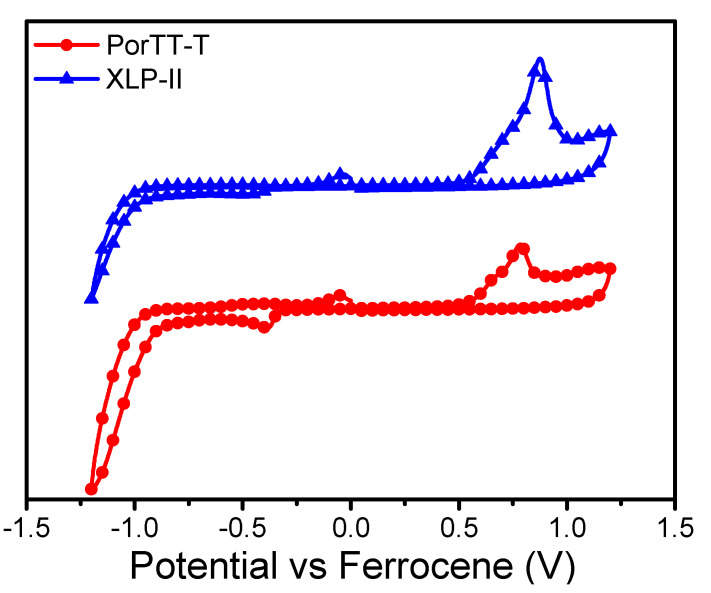
Cyclic voltammetry (CV) curve of PorTT-T flim measured in acetonitrile with 0.1 M tetrabutylammonium hexafluorophosphate (TBAPF_6_) calibrated with ferrocene/ferrocenium (Fc/Fc^+^) as an external reference.

Figure 4 shows the cyclic voltammetry (CV) curves of PorTT-T and XLP-II, and Table 3 presents the electrochemical data. We used ferrocene as the internal standard to correct the potential, and then used the tangent line from the peak onset of oxidation and reduction to obtain an oxidation potential (E_onset_^ox^) of 0.57 V and reduction potential (E_onset_^re^) of −0.92 V from the cyclic voltammetry results. Then, E_HOMO_ and E_LUMO_ values were estimated as −5.37 eV and 3.88 eV, respectively. E_HOMO_ was calculated from the onset oxidation potential according to the formula E_HOMO_ = −(E_onset_^ox^ + 4.8) eV, and E_LUMO_ was calculated according to the formula E_LUMO_ = −(4.8 + E_onset_^re^) eV [35]. Compared to XLP-II, both E_HOMO_ and E_LUMO_ values of PorTT-T were reduced. The decrease in E_LUMO_ was more pronounced, and the band gap of PorTT-T was narrower. According to the DFT results for the frontier molecular orbitals, the introduction of the S atom enhanced the electronegativity of the side chain, reduced the electron density in the porphyrin core, promoted the electron transfer on the main chain, and enhanced the end-group effect.

### 2.3. OSC Performance

**Figure 5:**
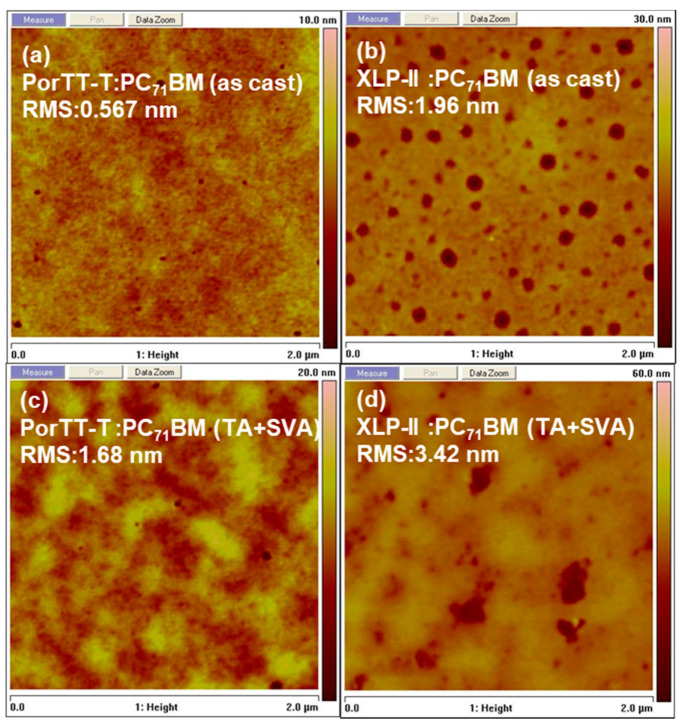
AFM topography images of active layer (D:A) blend films, (**a**,**b**) as-cast from chlorobenzene, and (**c**,**d**) after annealing at 100 °C for 10 min with treatment with CF (TA + SVA).

Atomic force microscopy (AFM) images for the phase, height, and roughness of the blend films of PorTT-T:PC_71_BM and XLP-II:PC_71_BM with different treatment modes are presented in Figure 5. Figure 5a–d respectively show their blend films without any treatment and the blend film after annealing at 100 °C for 10 min and treatment with chloroform. The root-mean-square roughness (RMS) values of the films of PorTT-T:PC_71_BM as-cast and processed with TA and with post-treatment with chloroform (CF) for 30 s were 0.477 nm and 1.68 nm, respectively. The root-mean-square roughness (RMS) values of the films of XLP-II:PC_71_BM as-cast and processed with TA and with post-treatment with CF for 30 s were 1.96 nm and 3.24 nm, respectively. The blend films with post-treatment displayed slightly higher RMS roughness, greater phase separation, and larger domain sizes compared to the PorTT-T:PC_71_BM blend films cast with chlorobenzene (CB). The post-treated blend films (PorTT-T:PC_71_BM) presented more uniform phase separation than the films without post-treatment. This trend is consistent with the properties of the materials examined in our previous work. More importantly, PorTT-T exhibited smaller roughness, less phase separation, and a smoother surface morphology than XLP-II for both post-treated and as-cast mixed films [33]. This indicates that the introduction of S atoms into the side chain led to the PorTT-TT:PC_71_BM blend films becoming more uniform and ordered than the XLP-II:PC_71_BM blend films.

**Figure 6:**
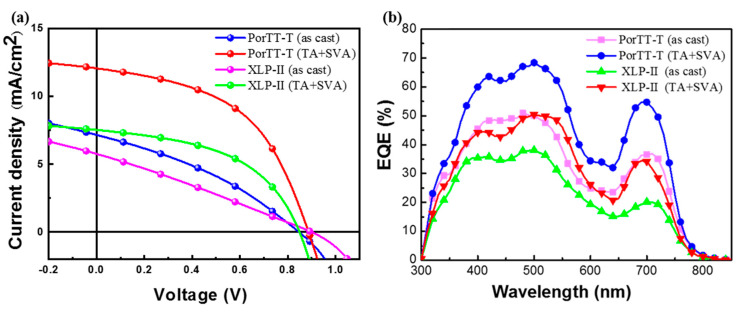
(**a**) Forward scanning current density–voltage (J–V) curves based on without any treatment (as-cast) and with 100 °C annealing for 10 min and CF vapor annealing for another 30s (TA + SVA) PorTT-T and XLP-II. (**b**) EQE spectra of donor (PorTT-T/XLP-II):PC_71_BM blend films in organic semiconductor devices.

Figure 6 presents the J–V curves (Figure 6a) and the corresponding external quantum efficiency (EQE) results of the devices for the active layer with different post-treatments. The film was exposed to chloroform vapor for 10 min after annealing in 100 °C. Table 4 presents the photovoltaic parameters of the devices with different post-processing characteristics for the materials. To enhance the device performance, we chose the use of a donor/acceptor mass ratio of 1:1.2 for the bulk blend films. We adopted different annealing temperatures, annealing times, solvent post-treatment, and solvent treatment times for material processing. During preparation, the device was subjected to 100 °C annealing for 8 min for multiple tests of the films, and the average values were then obtained for all of the performance parameters of the material. It was found that the open-circuit voltage of the PorTT-T was 0.662 V, the current density was 8.44 mAcm^−2^, the fill factor (FF) was 32.1%, and the photovoltaic power conversion efficiency was 1.98%. Next, for the annealed film, we used different solvents, namely dichloromethane (DCM), chloroform (CF), and carbon disulfide (CS_2_), for the treatment of the material after annealing. Device properties such as the open circuit voltage, current density, and fill factor were increased by the solvent treatment, with significant increases in the current density obtained for the DCM- and CF-processed materials to over 11 mAcm^−2^, and the PCE of the CF device reached 3.25%. It is important to note that while the current density of the devices using materials post-processed by CS_2_ did not change as much as for the other two solvents, the open-circuit voltage and fill factor were the most significantly improved, where that the PCE device reached 3.22%, second only to the device based on the CF-treated material. We then attempted to optimize the annealing time and CS_2_ solvent treatment time, but unfortunately did not find better conditions. By contrast, for the CF treatment, after increasing the annealing time the open-circuit voltage and filling factor of the devices obtained by CF post-annealing treatment also exceeded the values obtained for the CS_2_-treated films, while maintaining the high current density. In the process of further PorTT-T:PC_71_BM device optimization, we found that the changes in the annealing temperature, annealing time, and solvent processing time led to a small change in the fill factor of the devices but did not lead to a significant boost in device performance. Unfortunately, higher annealing temperature and longer post-processing times reduced the current density of the device. Therefore, we finally chose 100 °C annealing for 10 min and chloroform post-treatment for 40 s as the optimal conditions for device fabrication. The device fabricated using the film obtained in these conditions had open-circuit voltage, short-circuit current density, fill factor, and photovoltaic conversion efficiency values of 0.82 V, 11.13 mAcm^−2^, 49.7%, and 4.56%, respectively.

For a fair comparison with the previously investigated donor material (XLP-II), we used the ITO/PEDOT:PSS/D:A/poly[(9,9-bis(3′-((*N*,*N*-dimethyl)-*N*-ethylammonium)- propyl)-2,7-fluorene)-alt-2,7-fluorene)-alt-2,7-(9,9-dioctylfluorene)]dibromide (PFN-Br)/Al device architecture. Under the same conditions, the performance characteristics of the PorTT-T:PC_71_BM device using the film without any treatment were as follows: a PCE of 2.06% with a *V*_oc_ of 0.845 V, a short-circuit current (*J*_sc_) of 7.15 mAcm^−2^, and a fill factor (FF) of 0.341. After thermal annealing (TA) at 100 °C for 10 min, the PCE, *V*_oc_, *J*_sc_, and FF increased to 2.89%, 0.938 V, 9.15 mAcm^−2^, and 0.337, respectively. Then, post-treatment of the film with CF vapor annealing (SVA) for 30 s led to device performance improvement, with PCE, *V*_oc_, *J*_sc_, and FF values of 5.32%, 0.886 V, 12.03 mAcm^−2^, and 49.9%, respectively. Thus, regardless of the processing conditions, compared to XLP-II:PC_71_BM, a significantly improved PCE was obtained with PorTT-T:PC_71_BM. The short-circuit current density of the device showed the most pronounced change. The current density of PorTT-T increased by a factor of more than 1.6 for the optimized device. The results also show that the introduction of the S atom in the side chain has a beneficial effect on the short-circuit current density of the donor materials.

**Figure 7:**
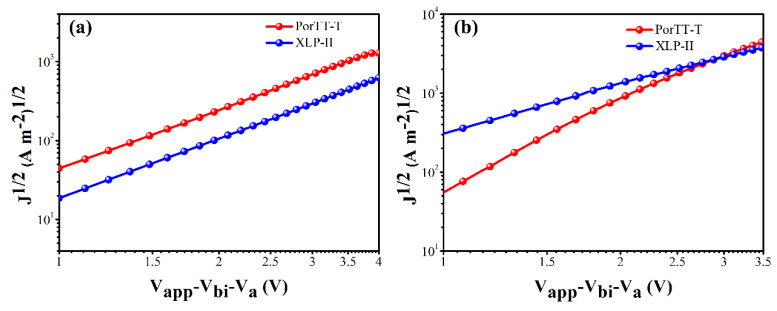
Mobility of excited charge carriers of PorTT-T and XLP-II. (**a**) Hole transport for an ITO/ZnO/D:A/PFN-Br/Al device and (**b**) electron transport for an ITO/PEDOT/D:A/MoO_3_/Al device.

To more fully understand the role of PorTT-T in the device, we measured the excited charge carrier mobility of the devices fabricated using the films obtained without any processing using the space-charge limiting current method [36]. Figure 7 shows the current–voltage curves for the measurement of hole (Figure 7a) and electron transport (Figure 7b). The electron mobility (μ_e_) of PorTT-T:PC_71_BM was 1.06 × 10^−4^ cm^2^ V^−1^ s^−1^, close to the value of 1.07 × 10^−4^ cm^2^ V^−1^ s^−1^ that we previously reported for XLP-II [33]. However, the corresponding hole mobility values (μ_h_) were 2.51 × 10^−5^ cm^2^ V^−1^ s^−1^ and 1.09 × 10^−5^ cm^2^ V^−1^ s^−1^, respectively. The corresponding μ_e_/μ_h_ ratios were 4.22 and 9.82, respectively. It was observed that the electron and hole transport of PorTT-T was more balanced than that of XLP-II, and the carrier separation was more pronounced. The small molecule PorTT-T with a thioalkyl side chain showed better performance than XLP-II. We believe that this is closely related to the introduction of S atoms on the side chain of PorTT-T that plays a positive role in carrier separation and transmission due to its enhancement of the intermolecular interactions that gives rise to a more ordered arrangement of the molecules [13].

## 3. Material and Method

### 3.1. Synthesis

The reagents used in the experiment were obtained from Dieckmannchem (Shenzhen, China). Dichloromethane (DCM), chloroform (CF), carbon disulfide (CS_2_), chlorobenzene (CB), and tetrahydrofuran (THF) were used after drying and vaporization in the laboratory (THF was dried with metal sodium and chloroform was dried by CaCl_2_). The other reagents were used directly with no further treatment. 2,5-*Bis*((2-ethylhexyl)oxy)-4-ethynylbenzaldehyde and Di(1H-pyrrol-2-yl)methane were synthesized according to a previous report [37]. As shown in Figure 1, a sulfur atom was inserted between the thieno [3,2-*b*] thiophene-porphyrin and the ethylhexyl chain, while the rest of the molecule was identical to XLP-II. The synthesis route is shown in Scheme S1, and follows previous reports [26,33,37]. A detailed description of the synthesis and characterizations is provided in the Supplementary Materials. The compounds were purified on a silica gel column, followed by Soxhlet extraction in acetone (24 h) to remove the impurities. Their chemical structures and purity were verified by nuclear magnetic resonance (NMR) spectrarecorded using a Bruker Ultrashield 400 Plus NMR spectrometer; Gas chromatography mass spectrometry (GC-Mass) and Matrix-Assisted Laser Desorption/Ionization Time of Flight Mass Spectrometry (MALDI-TOF MS) (Bruker Daltonic Inc., Beijing, China). The abbreviation of s, d, dd and m were defined the spike following as: s (singlet); d (doublet); dd (doublet of doublets); J (coupling constant); δ (shifts ppm). The abbreviation of reaction time: for hour is (h), for minutes is (min.) and for seconds is (s).

### 3.2. Characterizations

The UV-Visa absorption of solution and films of PorTT-T and XLP-II were measured with a UV-Cary 60 apparatus (Agilent Technologies Inc., Santa Clara, CA, USA) with a concentration of 1 × 10^−6^ mol/L chloroform solution, and the films were drop-coated on a quartz glass plate. The variable-temperature UV-Vis spectrum was obtained by UV-100 with a concentration of 1 × 10^−5^ mg/L in chlorobenzene. The cyclic voltammetry (CVs) of samples was measured with 0.1 M tetrabutylammonium hexafluorophosphate in acetonitrile (CH_3_CN) in a CHI660E electrochemical workstation (Shanghai Chenhua Instrument Co., Ltd., Shanghai, China), which was the supporting electrolyte, and ferrocene/ferrocenium (Fc/Fc+) represented the internal standard sample. Samples were dissolved in CF, and then deposited on the platinum disk working electrode to form a film. Ag/AgNO_3_ (0.01 M in CH_3_CN) was used as the reference electrode, and the Pt electrode was used as the auxiliary electrode. The potentials were internally calibrated using the ferrocene/ferrocenium of the (Fc/Fc+) redox couple, and the scanning rate was 50 mV/s. The morphology images were recorded in tapping mode using a Bruker Multimode 8 microscope (Bruker Corporation, Billerica, Germany) with TESP probes at 100 kV. The donor:PC_71_BM blend films (surface area: 2 × 2 μm^2^) were cast from chloroform solution. Density functional theory calculations were performed using the Gaussian 09 program package at the B3LYP/6-31G(d)* level of theory.

### 3.3. Device Fabrication

The OSC devices were fabricated according to literature reports [38] following the structure of indium tin oxide (ITO)/poly(3,4-ethylenedioxythiophene):poly(styrenesulfonate) (PEDOT:PSS)/poly[(9,9-bis(3′-((*N*,*N*-dimethyl)-*N*-ethylammonium)-propyl)-2,7-fluorene)-alt-2,7-fl-uorene)-alt-2,7-(9,9-dioctylfluorene)]dibromide (PFN-Br)/aluminum (Al), or ITO/PEDOT:PSS/D:PC_71_BM/calcium (Ca)/Al. The area of the device was 0.06 cm^2^. PEDOT:PSS (Clevios PVP Al 4083) was obtained from H.C. Starck Germany (Munich, Germany). PEDOT:PSS was coated on ITO glass (Shenzhen Huayu United Technology Co., LTD, Shenzhen, China, 15 ohm/sq) with 4000 revolutions per minute for 40 s, and the thickness was about 40 nm. Then, annealing was performed using a heating panel at 140 °C for 20 min, under a glove box with nitrogen. The thickness of the activity layer was about 100 nm. The activity layer of the donor:PC_71_BM (ratio fixed as 1:1.2 by weight) solution was prepared, where the solvent was the mixture solvent of chlorobenzene (CB, 8 mg/mL) and pyridine (3 vol%). Then, it was spin-coating onto the PEDOT:PSS and ITO substrates, and then treated with different methods. Finally, the deposition of Ca (20 nm) or PFN-Br (5 nm) and Al electrodes of 100 nm was performed using vacuum evaporation. The thickness of the active layer of the device was measured using a Veeco Dektak 150 surface profiler (USA VIECO Precision Instrument Co., Ltd., Shanghai, China). The current density–voltage (J–V) characteristics of the organic solar cells were determined using a programmable Keithley 2400 source measurement unit under simulated solar light (AM 1.5 G) (DM40S3, SAN-EI ELECTRIC CO., LTD. Osaka, Japan), and the devices were masked during measurement. The spectral response was measured with a DSR100UV-B spectrometer with a SR830 lock-in amplifier (Beijing Zhuoli Hanguang Instrument Co., Ltd., Beijing, China). A calibrated Si photodiode was used as a reference before each measurement.

## 4. Conclusions

To explore the effect of the introduction of heteroatoms on the properties of porphyrin materials, we modified the XLP-II molecule synthesized in previous work to obtain a new small-molecule material (PorTT-T). An S atom was added to the contact position of the porphyrin side chain, changing the original alkyl branched chain into a sulfhydryl branched chain, and its conjugated bridge and end groups were 1,4-*bis*((2-ethylhexyl)oxy)benzene and 3-ethylrhodanine, respectively. We characterized the basic properties of PorTT-T and compared its performance to that of XLP-II. An examination of the UV-visible light absorption at the temperatures down from 90 to 20 °C showed that the absorption spectrum of PorTT-T was redshifted by more than 10 nm, indicating that molecules were assembled with decreasing temperature. A comparison of the spectral absorption of the films after different treatments showed that the absorption of PorTT-T displayed a clear redshift after temperature annealing and solvent vapor annealing. DFT calculations and cyclic voltammetry results showed that the introduction of the S atom on the side chain group led to the lowering of the HOMO and LUMO positions and narrowed the band gap. The AFM phase images showed that the surface roughness was smaller, and the surface molecular arrangement was more ordered. Device characterization results showed that an improved open-circuit voltage of 0.886 eV, short-circuit current of 12.03 mAcm^−2^, and fill factor of 0.499 were obtained, which, together with a higher external quantum efficiency, led to a high PCE of 5.32%. Meanwhile, compared to XLP-II, a higher hole mobility of 2.51 × 10^−5^ m^2^ V^−1^ s^−1^ and a better μ_e_/μ_h_ ratio of 4.22 were obtained for PorTT. These results show that the properties of PorTT-T were significantly improved by the introduction of S atoms on the side chain of porphyrin. Of course, much work still remains to be done for this system. The fill factor is a key factor affecting the photovoltaic conversion efficiency of the PorTT-T devices. We hope to improve the FF of these kind of small molecular materials based on porphyrin in our follow-up research. While in this work we only modified the side group using the S atom, in future research we will seek to introduce other heteroatoms or groups on the side chain in order to obtain high-performance organic semiconductor materials for organic photoelectric devices.

## Figures and Tables

**Table 1:** The data of UV-Vis absorption of PorTT-T and XLP-II in chlorobenzene from 20 to 90 °C (numbers in brackets are the absorption coefficients).

Temperature (°C)	PorTT-T		XLP-II	
	Q-Band (nm) (L/mol·cm)	B-Band (nm) (L/mol·cm)	Q-Band (nm) (L/mol·cm)	B-Band (nm) (L/mol·cm)
20	493 (1.70), 508 (1.65)	681 (0.75)	481 (1.68), 508 (1.23)	681 (0.60)
30	492 (1.69), 506 (1.64)	679 (0.74)	481 (1.67), 505 (1.22)	679 (0.58)
40	491 (1.68), 504 (1.62)	678 (0.73)	480 (1.67), 505 (1.22)	678 (0.56)
50	490 (1.68), 503 (1.61)	677 (0.71)	480 (1.67), 502 (1.21)	677 (0.55)
70	489 (1.67), 498 (1.61)	675 (0.67)	480 (1.51), 505 (1.16)	675 (0.51)
90	488 (1.61)	673 (0.60)	484 (1.26)	674 (0.45)

**Table 2:** The data of optical absorption and the optical band gap of PorTT-T and XLP-II.

Samples	λ_max_ (Solution) (nm)	λ_max_ (Film) (nm)	λ_onset-edge_ (nm)	E_g_^opt^ (nm)
PorTT-T	492, 504, 695	524,694	772	1.60
XLP-II	483, 502, 692	505,690	770	1.61

Notes: λ_onset-edge_ means the edge onset redshift of films; E_g_^opt^ = 1024/λ_onset-edge_.

**Table 3:** The CV data and the DFT calculation results.

Samples	E_onset_^ox^ (V)	E_HOMO_ (eV)	E_LUMO_ (eV)	E_onset_^Re^ (V)	E_HOMO_^cal^ (eV)	E_gap_^cal^ (eV)	E^cal^_LUMO_ (eV)
PorTT-T	0.57	−5.37	−3.88	−0.92	−5.14	1.96	−3.10
XLP-II	0.53	−5.33	−3.78	−1.02	−5.06	2.03	−3.03

Note: E_HOMO_ = −(E_onset_^ox^ + 4.8) eV and E_LUMO_ = −(4.8 + E_onset_^re^) eV; E_HOMO_^cal^ and E_LUMO_^cal^ were calculated by DFT.

**Table 4:** Photovoltaic performance of bulk heterojunction solar cells based on donor (PorTT-T/XLP-II):PC_71_BM with weight ratios of 1:1.2 cast from CB and different post-treatments using PFN-Br/Al and LiF/Ca/Al as the cathode under AM1.5G illumination (100 mW·cm^−2^).

Donors	The Cathode	TA (Temperature/Time)	SVA (Solvent/Time)	V_oc_ (V)	J_sc_ (mAcm^−2^)	FF (%)	PCE (%)
PorTT-T:PC_71_BM	PFN-Br/Al	No	No	0.845	7.15	34.1	2.06
	PFN-Br/Al	100 °C/10 min	No	0.938	9.15	33.7	2.89
	PFN-Br/Al	100 °C/10 min	CF/30 s	0.886	12.03	49.9	5.32
	LiF/Ca/Al	100 °C/8 min	DCM/20 s	0.662	8.44	32.1	1.98
	LiF/Ca/Al	100 °C/8 min	DCM/20 s	0.68	11.04	34.55	2.6
	LiF/Ca/Al	100 °C/8 min	CF/20 s	0.75	11.32	38.45	3.25
	LiF/Ca/Al	100 °C/8 min	CS_2_/20 s	0.76	10.64	39.9	3.22
	LiF/Ca/Al	100 °C/10 min	CS_2_/20 s	0.74	10.27	39.35	2.98
	LiF/Ca/Al	100 °C/10 min	CS_2_/30 s	0.74	9.2	37.8	2.58
	LiF/Ca/Al	100 °C/10 min	CS_2_/40 s	0.77	7.85	36.05	2.18
	LiF/Ca/Al	100 °C/10 min	CF/20 s	0.80	11.27	44.5	3.99
	LiF/Ca/Al	100 °C/10 min	CF/30 s	0.82	11.16	47.28	4.30
	LiF/Ca/Al	100 °C/10 min	CF/40 s	0.82	11.13	49.7	4.56
	LiF/Ca/Al	100 °C/10 min	CF/50 s	0.83	10.44	51.55	4.49
	LiF/Ca/Al	100 °C/15 min	CF/40 s	0.83	10.72	50.2	4.48
	LiF/Ca/Al	90 °C/10 min	CF/40 s	0.84	10.90	50.82	4.64
	LiF/Ca/Al	110 °C/10 min	CF/40 s	0.82	10.85	50.2	4.47
XLP-II:PC_71_BM	PFN-Br/Al	No	No	0.894	5.76	27.2	1.40
	PFN-Br/Al	100 °C/10 min	No	0.863	8.03	32.6	2.26
	PFN-Br/Al	100 °C/10 min	CF/30s	0.847	7.51	49.3	3.14

## Data Availability

Not applicable.

## Supplementary Materials

The supplementary materials are available online. Scheme S1: Synthesis routes of PorTT-T and XLP-II, Figure S1: The 1H-NMR of PorTT-T, Figure S2: The 1H-NMR of XPL-II, Figure S3: The 13C-NMR of PorTT-T, Figure S4: The 13C-NMR of XLP-II, Figure S5: The schematic diagram of optimized structure of PorTT-T and XLP-II obtained by DFT calculations.

## Sample Availability

Samples of the compounds are available from the authors.
