# Supplementary material for: Effects of Side-Chain Engineering with the S Atom in Thieno[3,2-b]thiophene-porphyrin to Obtain Small-Molecule Donor Materials for Organic Solar Cells
Source: Molecules. 2021 Oct 11;26(20):6134. doi: 10.3390/molecules26206134 (PMC8538340; doi:10.3390/molecules26206134)
Supplement: Supplementary file 1 [file molecules-26-06134-s001.zip › molecules-1261259-supplementary.pdf]

molecules

MDPI

Electronic Supporting Information

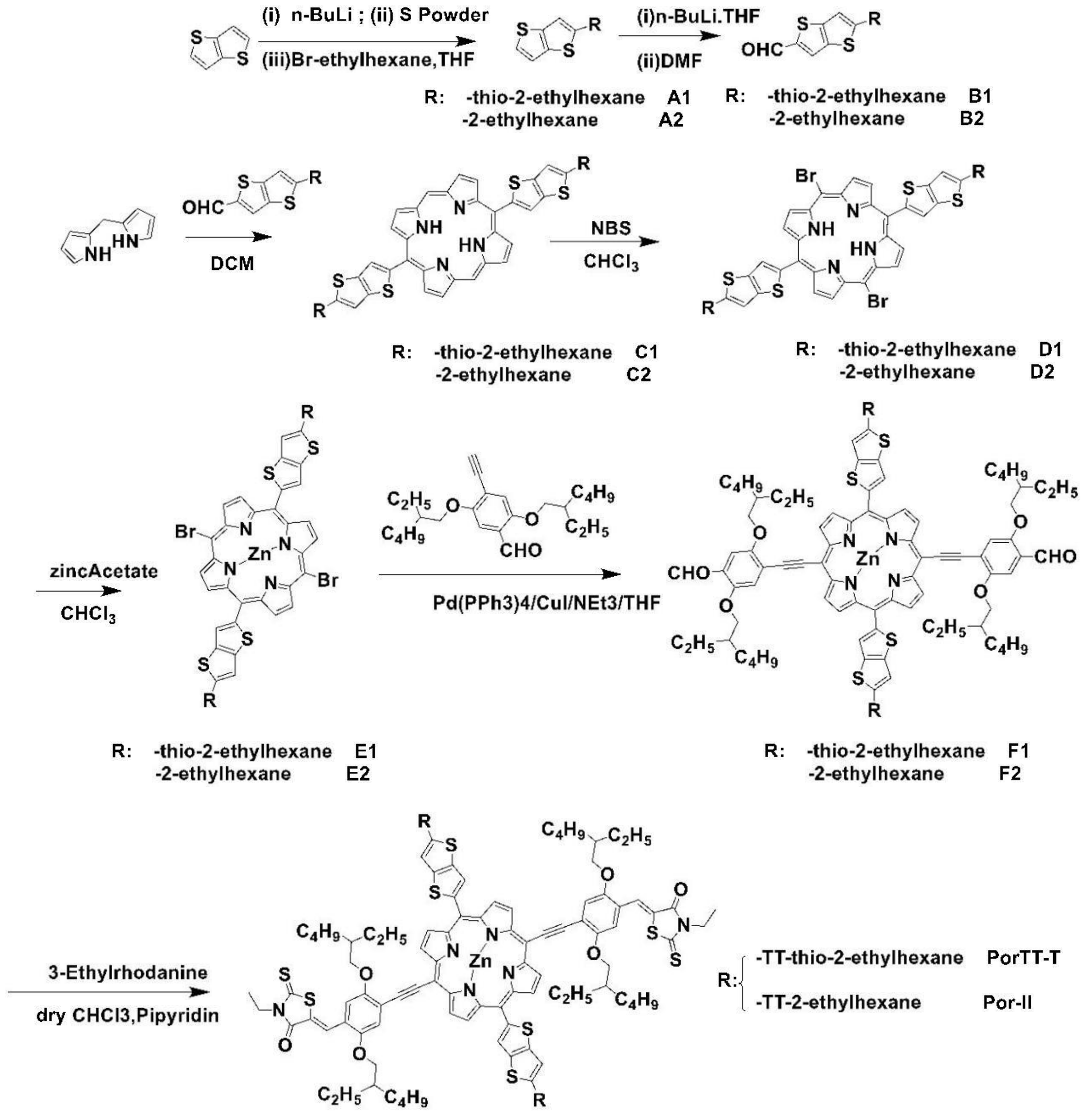

Scheme S1. Synthesis routes of PorTT-T and XLP-II

2-((2-Ethylhexyl)thio)thieno[3,2-b]thiophene (A1)

Under the protection of argon, n-butyllithium (n-BuLi) (2.5 M, 14.26 mL, 35.6 mmol) was dropwise added to thieno[2,3-b]thiophene (5.00g, 35.60 mmol) in dry tetrahydrofuran THF (20 mL) at −78 °C over 1 h. The Sulphur Powder (0.92 , 28.48mmol) was added. After being stirred for 1 h, the mixture was warmed to room temperature and stirred for another 0.5 h.3-(bromomethyl)heptane (5.50 g, 28.48 mmol), then was added into the mixture at −78 °C in one portion, and the mixture was warmed to room temperature and stirred for 12 h. Subsequently, the mixture was poured into water and extracted with petroleum ether (20 mL × 2). The organic layer was washed with water and dried

over anhydrous Na_2SO_4 for 3 h. After removal of solvent, the crude product was purified by silica gel using petroleum ether as eluent to afford compound 2a as a colorless liquid (2.40 g, 41%).

$_1$H NMR (400 MHz, $CDCl_3$) δ 7.41 (d,J = 2.1 Hz, 1H), 7.32 (s, 1H), 7.20 (d, J = 2.0 Hz, 1H), 2.88 (d, J = 3.6 Hz, 2H), 1.46 (m, 5H), 1.28 (m, 4H),0.99 – 0.73 (m, 6H).

GC-Mass: ($C_{14}H_{20}S_3$), calculated: 284.4940, found: 284.0730.

2-(2- Ethylhexyl)thieno[3,2-b]thiophene(A2)

Under the protection of argon, n-butyllithium (2.5 M, 14.26 mL, 35.6 mmol)was dropwise added to thieno[2,3-b]thiophene (5.00g, 35.60 mmol) in dry THF (20 mL) at −78 °C over 1 h. and then stirred for another 0.5 h. 3-(bromomethyl)heptane (5.50 g, 28.48 mmol), then was added into the mixture at −78 °C in one portion, and the mixture was warmed to room temperature and stirred for 12 h. the rest procedures was following to A1, with the yield of 60%. $_1$H NMR (400 MHz, $CDCl_3$) δ 7.27 (d, J = 5.9 Hz, 1H), 7.18 (d, J = 5.2 Hz, 1H), 6.94 (s, 1H), 2.81 (d, J = 6.8 Hz, 2H), 1.62 (d, J = 4.9 Hz, 1H), 1.57 – 1.05 (m, 8H), 0.90 (dd, J = 10.0, 4.7 Hz, 6H). GC-Mass:（$C_{14}H_{20}S_2$）m/z: calcd for 252.4340, found: 252.1010.

5-((2-Ethylhexyl)thio)thieno[3,2-b]thiophene-2-carbaldehyde(B1) and

5- Ethylhexylthieno[3,2-b]thiophene-2-carbaldehyde (B2)

A1/A2 (8.4 mmol) was weighed into a three-necked -bottomed flask (100 ml) under a N_2 atmosphere, and then anhydrous THF (40 ml) was added to the reaction flask, which was then cooled down to -78 °C by using a dry ice-acetone bath. At this point, an n-BuLi solution (6.72 ml, 16.8 mmol, 2.5 M) was added dropwise to the flask and the mixture was stirred for 1 Hour. The solution was slowly warmed to 0 °C and stirred for another 1 h. At which time a precipitate was formed, After cooling the flask to -78 °C, N-formylpiperidine (1.87 ml ,16.80 mmol) was added dropwise with a syringe where upon the reaction mixture turned clear, the cooling bath was removed and the reaction mixture was stirred overnight at room temperate. The reaction was quenched with water, and cooled down to 0°C. A sufficient amount of 3 M hydrochloricacid (HCl) aqueous solution was added to the reaction mixture to mark the solution slightly acidic and solution was extracted with dichloromethane.

B1:$_1$H NMR (400 MHz,$CDCl_3$): δ 9.97 (s, 1H), 7.82 (s, J = 2.0 Hz, 1H), 7.26 (s, 1H), 2.97 (d, J = 3.0Hz,2H), 1.61 (m, 1H), 1.48–1.28(m, 9H), 0.89–0.88 (m, 6H). GC-Mass:（$C_{15}H_{20}OS_3$）m/z: calcd for 312.5040, found:312.0680.

; **B2:**$_1$H NMR (400 MHz, $CDCl_3$) δ 9.92 (s, 1H), 7.85 (s, 1H), 7.01 (s, 1H), 2.85(d, J = 6.9 Hz, 2H), 1.85 – 1.61 (m, 1H), 1.46 – 1.07 (m, 8H), 0.95 – 0.81 (m, 6H). GC-Mass:（$C_{15}H_{20}OS_2$）m/z: calcd for 280.4440, found: 280.0960.

5,15-Bis(5- (2-ethylhexyl)thio)thieno[3,2-b]thiophen-2-yl)porphyrin (C1) and 5,15-Bis(5-(2-ethylhexyl)thieno[3,2-b]thiophen-2-yl)porphyrin (C2)

A solution of dipyrromethane(DPM) (3 g, 21 mmol) and carbaldehyde (**B1 and B2**) (21 mmol) in CH_2Cl_2 (1.5 L) was degassed by bubbling with nitrogen for 30 min. Then trifluoroacetic acid (TFA) (145 μL, 2.0 mmol) was added. After the solution was stirred overnight at room temperature under nitrogen, 2,3-dichloro-5,6-dicyanobenzoquinone (DDQ) (7.28 g, 32 mmol) was added, and the reaction mixture was stirred for a further 2 h. Triethylamine(NEt_3) (5 mL) was added. The solvent was evaporated and the crude product was purified by silica chromatography using CH_2Cl_2/n-hexane (1:4) as the eluent, to give a purple product. **C1**:$_1$H NMR (400 MHz, $CDCl_3$) δ 10.26 (s, 2H), 9.35 (t,J = 2.9 Hz, 8H), 8.01 (d,J = 0.6 Hz, 2H), 7.57 (d,J = 0.6 Hz, 2H), 7.25 (s, 2H), 3.07 (d,J = 6.4 Hz, 4H), 1.85 – 1.71 (m, 2H), 1.69 – 1.44 (m, 10H), 1.43 – 1.26 (m, 8H), 1.06 – 0.93 (m, 12H), -3.06 (s, 2H), MALDI-TOF MS（$C_{48}H_{50}N_4S_6$）m/z: calcd for 875.3160, found: 875.3149.;

C2:$_1$H NMR (400 MHz, $CDCl_3$) δ10.32 (s, 2H), 9.42 (dd, J = 11.8, 4.6 Hz, 8H), 8.06 (s, 2H), 7.24 (s, 2H), 3.05 (d, J = 5.9 Hz, 4H), 1.97–1.74 (m, 2H),1.52 (dd, J = 10.5, 6.9 Hz, 4H), 1.48–1.32 (m, 12H),1.08–0.99 (m, 12H), -2.95 (s, 2H). MALDI-TOF MS（$C_{48}H_{50}N_4S_4$）m/z: calcd for 811.1960, found: 811.1962.

5,15-Dibromo-10,20-bis(5-((2-ethylhexyl)thio)thieno[3,2-b]thiophen-2-yl)porphyrin (D1)

Solution of **C1** (1.00 mmol), NBS (373 mg, 2.10 mmol) and pyridine (0.1 mL) in CH_2Cl_2 (200 mL) was stirred at 0 °C for 30 min. Acetone (5 mL) was added, and the solvent was removed in vacuo. The residue was purified via chromatography on gel using CH_2Cl_2/n-hexane (1:4) as the eluent to give the product. **D1**: $_1$H NMR (400 MHz, $CDCl_3$) δ 9.65(d, J = 2.4 Hz, 4H), 9.16 (d, J = 2.2 Hz, 4H), 7.93 (d, J = 0.2Hz,2H), 7.54 (s, J = 0.2 Hz, 2H), 3.06 (d, J = 3.2 Hz, 4H), 1.801 – 1.73 (m, 2H), 1.43-0.98 (s, 16H), 0.82 (m, 12H), -3.05 (s,2H); MALDI-TOF MS（$C_{48}H_{48}Br_2N_4S_6$）m/z: calcd for 1033.1080, found:1032.1076.

5,15-Dibromo-10,20-bis(5-(2-ethylhexyl)thieno[3,2-b]thiophen-2-yl)porphyrin (D2)

Solution of **C2** (1.00 mmol), NBS (373 mg, 2.10 mmol) and the others same with D1,abttained pure product **D2**: $_1$H NMR (400 MHz, $CDCl_3$) δ 9.59 (d, J = 4.3 Hz, 4H), 9.17 (d, J = 4.3 Hz, 4H), 7.97 (s, 2H), 7.20 (s, 2H), 3.03 (d, J = 6.5 Hz, 4H), 1.82 (s, 2H), 1.59 – 1.31 (m, 16H), 1.12 – 0.82 (m, 12H), -2.65 (s, 2H). MALDI-TOF MS（$C_{48}H_{48}Br_2N_4S_4$）m/z: calcd for 968.9880, found: 968.9869.

5,15-Dibromo-10,20-bis(5-((2-ethylhexyl)thio)thieno[3,2-b]thiophen-2-yl)zincporphyrin (E1) and 5,15-Dibromo-10,20-bis(5-(2-ethylhexyl)thieno[3,2-b]thiophen-2-yl)zincporphyrin (E2)

equal molar amounts of E1 and E2 (1mol) were placed in two reaction flask respectively, and then 5 mmol of zinc acetate (1.095 g) were added , refluxed in $CHCl_3$ (200 mL) for overnight. The solvent

was removed and the residue was purified via chromatography on silica gel using CH_2Cl_2/n-hexane (1:4) as the eluent to give the product over 70% yield. **E1**: ^{1}H NMR (400 MHz, $CDCl_3$) δ 9.65 (d, J = 2.4 Hz, 4H), 9.16 (d, J = 2.2 Hz, 4H), 7.93 (s, 2H), 7.54 (s, 2H), 3.06 (d, J = 3.2 Hz, 4H), 1.78 – 1.73 (m, 2H), 1.64 – 1.20 (m, 16H), 1.05 – 0.91 (m, 12H). MALDI-TOF MS ($C_{48}H_{46}Br_2N_4S_6Zn$) m/z: calcd for 1096.4720, found: 1094.4715. **E2**: ^{1}H NMR (400 MHz, $CDCl_3$) δ 9.64 (d, J = 4.6 Hz, 4H), 9.21(d, J = 4.6 Hz, 4H), 7.93 (s, 2H), 7.19 (s, 2H), 3.02 (d, J = 5.4 Hz, 4H), 1.82 (s, 2H), 1.55 – 1.30 (m, 16H), 1.03 (dd, J = 16.4, 9.1 Hz, 12H). MALDI-TOF MS（$C_{48}H_{46}Br_2N_4S_4Zn$）m/z: calcd for 1032.3520, found: 1030.3507.

5,15-Bis(5-((2-ethylhexyl)thio)thieno[3,2-b]thiophen-2-yl)-10,20-(2,5-bis((2-ethylhexyl)oxy)-4-ethynylbenzaldehyde)zincporphyrin (F1) and 5,15-Bis(5-(2-ethylhexyl)thieno[3,2-b]thiophen-2-yl)-10,20-(2,5-bis((2-ethylhexyl)oxy)-4-ethynylbenzaldehyde)zincporphyrin (F2)

4-Ethynyl-2,5-bis(2-ethylhexyl)oxy)benzaldehyde (2.1 eq) and equal molar amounts of **E1** and **E2** (1mol) were placed in two reaction flask respectively. (150 mmol) were added to a two round necks bottom flask (100 ml), removed the oxygen and put the nitrogen（N_2）, repeat there times. Then 20 ml of dry THF and 10 ml of dry triethylamine was injected by syringe. Stirred for 10 mins, then $Pd(PPh_3)_4$ (60 mg, 0.053 mmol) was added, and then CuI (10 mg, 0.053 mmol) was added underN$_2$ atmosphere for10 min. The reaction was stirred at 60 °C for 24 h. The completion of the reaction was monitored by TLC. The solvent was removed by rotary evaporation. The residue was purified by column chromatography using CH_2Cl_2/n-hexanes = 1/2 as eluent to give the compound with yield of 78 %. **F1:**^{1}H NMR (400 MHz, $CDCl_3$) δ 10.48 (s, 2H), 9.76 (d, J = 4.4 Hz, 4H), 9.18 (d, J = 4.6 Hz, 4H), 8.00 (s, 2H), 7.57 (s, 2H), 7.48 (d, J = 10.3 Hz, 4H), 4.13 (dd, J = 28.0, 5.5 Hz, 8H), 3.08 (d, J = 6.4 Hz, 4H), 2.24 – 1.98 (m, 2H), 1.87 – 1.10 (m, 52H), 1.07 – 0.81 (m, 30H), 0.75 (t, J = 7.3 Hz, 6H). MALDI-TOF MS（$C_{98}H_{120}N_4O_6S_6Zn$）m/z: calcd for 1704.8000, found: 1708.7991. **F2:**^{1}H NMR (400 MHz, $CDCl_3$) δ 10.55 (s, 2H), 9.79 (d, J = 2.4 Hz, 4H), 9.21 (d, J = 2.4 Hz, 4H), 7.97 (s, 2H), 7.57 (s, 2H), 7.52 (s, 2H), 7.21 (s, 2H), 4.17 (dd, J = 8.0, 1.6 Hz, 8H), 3.03 (d, J = 3.2 Hz, 4H), 2.20 – 2.16 (m, 2H), 1.83 – 1.78 (m, 4H), 1.71 – 1.25 (m, 48H), 1.05 – 0.81 (m, 30H), 0.75 (t, J = 7.3 Hz, 6H). MALDI-TOF MS（$C_{98}H_{120}N_4O_6S_4Zn$）m/z: calcd for 1643.6800, found: 1641.6791.

Final products

Equal molar amounts of F1 and F2 were placed in two reaction flask respectively. Equal amounts of 3-ethyl circotannin were added, 2 drops of piperidine and 30ml of dry chloroform were followed, and stirring and refluxing for overnight. After the raw material used up, stop heat and remove the

solvent, washed with water, separated the crude products with chromatographic and recrystallized to abttained pure products, with a yield about 70%.**PorTT-T**: $_1$H NMR (400 MHz, $CDCl_3$) δ 9.69 (s, J = 4.6 Hz, 4H), 9.12 (d, J = 4.6 Hz, 4H), 8.17 (d, J = 6.8 Hz, 2H), 7.98 (s, 2H), 7.61 (s, 2H), 7.43 (d, J = 4.9 Hz, 2H), 6.95 (d, J = 8.0 Hz, 2H), 4.22 – 4.01 (m, 12H), 3.07 (d, J = 6.4 Hz, 4H), 2.16 (dd, J = 18.8, 12.9 Hz, 6H), 2.15 – 1.13 (m, 48H), 1.09 – 0.67 (m, 42H). MALDI-TOF MS（$C_{108}H_{130}N_6O_6S_{10}Zn$）m/z: calcd for 1994.2440, found: 1994.2432; **XLP-II**: $_1$H NMR (400 MHz, $CDCl_3$) δ 9.75 (s, J = 2.2 Hz, 4H), 9.19 (d, J = 2.2 Hz, 4H), 8.27 (s, 4H), 7.98 (s, 2H) , 7.43 (s, 2H), 7.25 (s, 2H), 4.25 – 4.12 (m, 12H), 3.03 (d, J = 3.4 Hz, 4H), 2.15 (dd, J = 18.8, 12.9 Hz, 6H), 2.15 – 1.13 (m, 48H), 1.04– 0.77 (m, 42H). MALDI-TOF MS（$C_{108}H_{130}N_6O_6S_8Zn$）m/z: calcd for 1930.1240, found: 1930.1213.

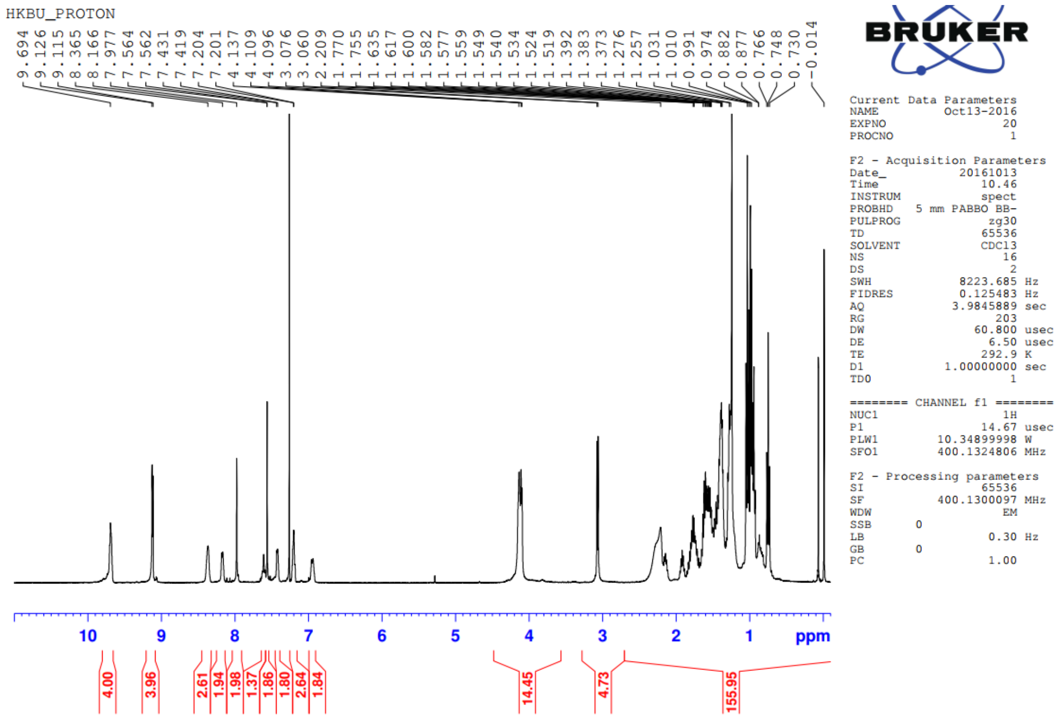

Figure S1. The ^{1}H-NMR of PorTT-T.

\

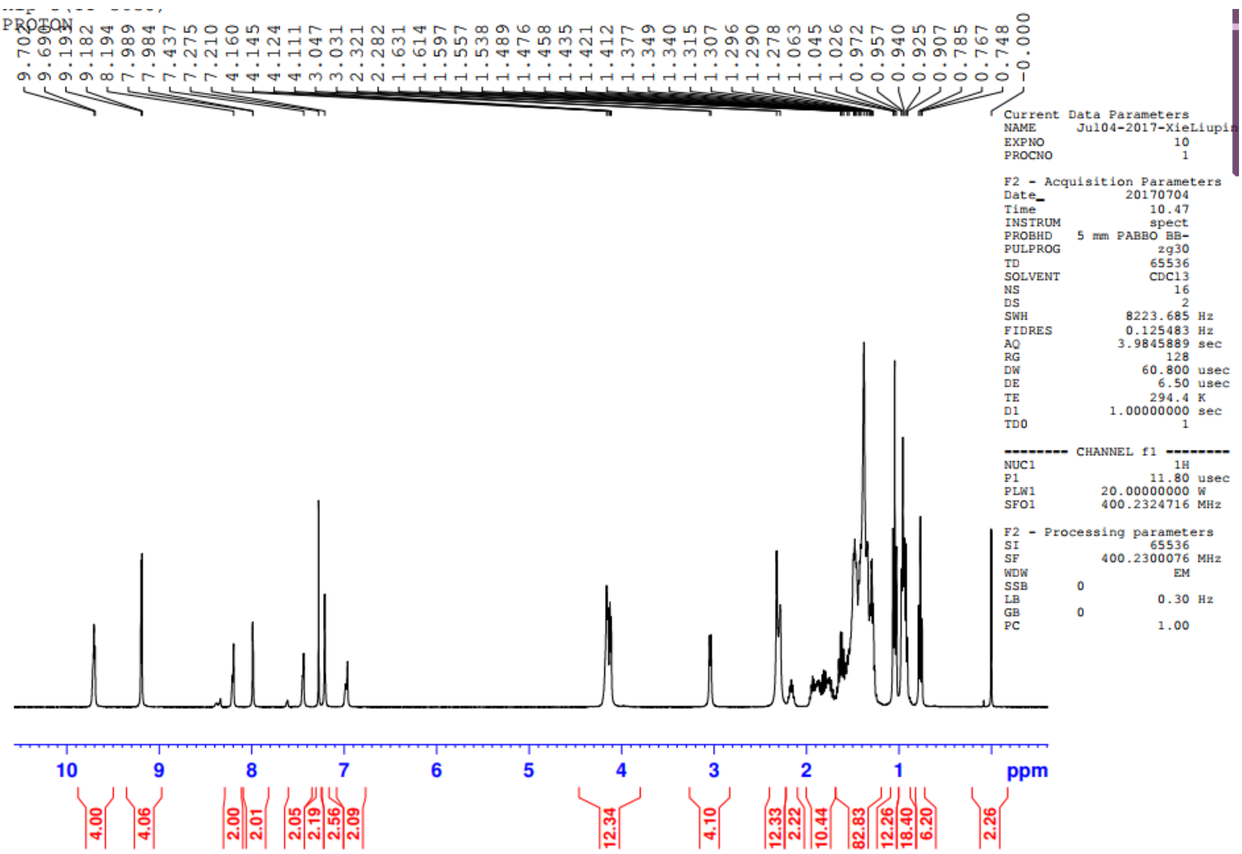

Figure S2. The ^{1}H-NMR of XPL-II.

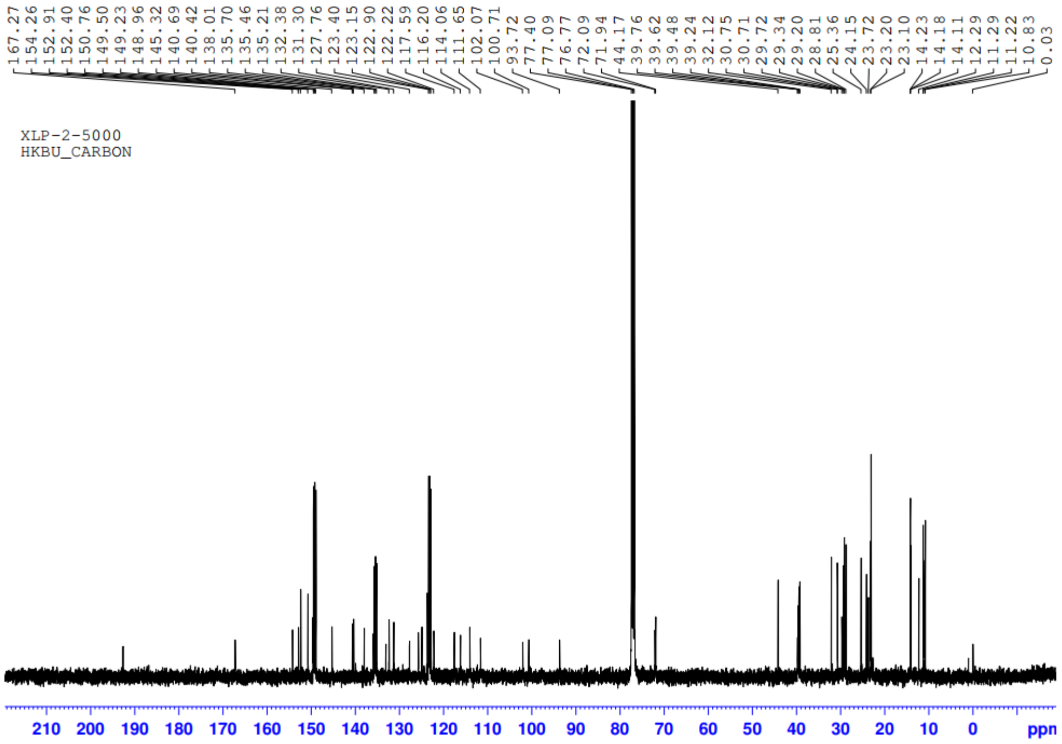

Figure S3. The ^{13}C-NMR of PorTT-T.

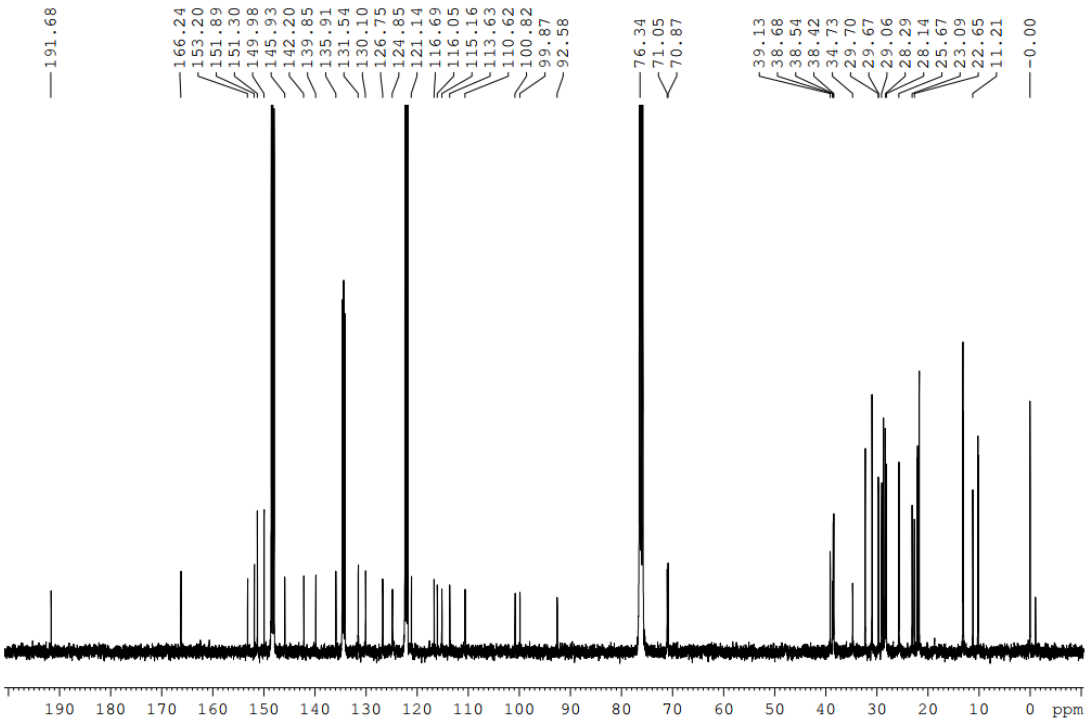

Figure S4. The ^{13}C-NMR of XLP-II.

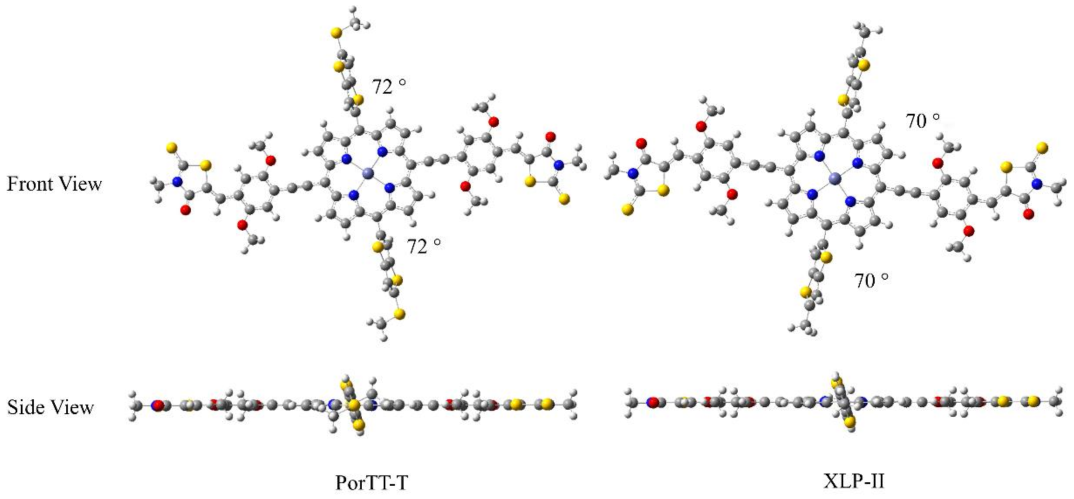

Figure S5. The schematic diagram of optimized structure of PorTT-T and XLP-II obtained by DFT calculations.
